# Conducting head-mounted eye-tracking research with young children with autism and children with increased likelihood of later autism diagnosis

**DOI:** 10.1186/s11689-024-09524-1

**Published:** 2024-03-04

**Authors:** E. Perkovich, A. Laakman, S. Mire, H. Yoshida

**Affiliations:** 1 University of Houston, Houston, TX USA; 2 Baylor University, Waco, TX USA

**Keywords:** Autism spectrum disorder, Attention, Social cognition, Social behavior, Eye-tracking

## Abstract

**Background:**

Over the past years, researchers have been using head-mounted eye-tracking systems to study young children’s gaze behaviors in everyday activities through which children learn about the world. This method has great potential to further our understanding of how millisecond-level gaze behaviors create multisensory experiences and fluctuate around social environments. While this line of work can yield insight into early perceptual experiences and potential learning mechanisms, the majority of the work is exclusively conducted with typically-developing children. Sensory sensitivities, social-communication difficulties, and challenging behaviors (e.g., disruption, elopement) are common among children with developmental disorders, and they may represent potential methodological challenges for collecting high-quality data.

**Results:**

In this paper, we describe our research practices of using head-mounted eye trackers with 41 autistic children and 17 children with increased likelihood of later autism diagnosis without auditory or visual impairments, including those who are minimally or nonspeaking and/or have intellectual disabilities. The success rate in gathering data among children with autism was 92.68%. 3 of 41 children failed to complete the play-session, resulting in an 86.36% success rate among 1–4-year-olds and a 100.00% success rate among 5–8-year-olds. 1 of 17 children with increased likelihood of later autism diagnosis failed to complete the play-session, resulting in a success rate of 94.11%. There were numerous “challenging” behaviors relevant to the method. The most common challenging behaviors included taking the eye-tracking device off, elopement, and becoming distressed. Overall, among children with autism, 88.8% of 1–4-year-olds and 29.4% of 5–8-year-olds exhibited at least one challenging behavior.

**Conclusions:**

Research capitalizing on this methodology has the potential to reveal early, socially-mediated gaze behaviors that are relevant for autism screening, diagnosis, and intervention purposes. We hope that our efforts in documenting our study methodology will help researchers and clinicians effectively study early naturally-occuring gaze behaviors of children during non-experimental contexts across the spectrum and other developmental disabilities using head-mounted eye-tracking. Ultimately, such applications may increase the generalizability of results, better reflect the diversity of individual characteristics, and offer new ways in which this method can contribute to the field.

## Introduction

There is growing interest in the study of gaze behaviors among toddlers and young children with autism for improving early screening and diagnosis processes [10, 14, 21]. Autism is a heterogeneous neurodevelopmental condition characterized by patterns of social-communication difficulties and restricted and repetitive behaviors [2], with an average diagnosis age of 4 years for children in the United States [28]. Early intervention is often viewed as critical to maximizing long-term outcomes, but access to services typically requires diagnosis or identification. Unfortunately, long waitlists, high costs, and lack of trained clinicians have contributed to later ages of diagnosis among children from racially, ethnically, linguistically, socioeconomically, and regionally marginalized communities [8, 31]. One potential mechanism to overcome such disparities is through access to objective and efficient screening, which may open the door to diagnosis and/or intervention, and early gaze behaviors have been proposed as a potential bio-behavioral marker that can help identify children likely to be diagnosed with autism in the future [14, 15, 21].

Despite the potential utility of gaze behaviors in identifying early risk and/or later diagnosis with autism, eye-tracking studies involving this population have traditionally used computer-mounted eye-tracking devices during computer-based tasks, which lack social context, restrict movement, and are more susceptible to data loss in cases where the child looks away from the computer screen [39]. Among typically-developing infants, literature has well-documented that early attention is a critical component of effective learning experiences and that early social interaction is vital for such attention development (e.g., the more contingent our early interactions, the better we can pay attention alone later on). Therefore, tracking attentional behaviors during social interaction among atypical populations poses interesting research questions despite the proposed methodological limitations. This has contributed to our lack of understanding about whether or not (and if so, how) parental input is relevant to atypical attention experiences—creating a significant gap in our understanding of the emergence of atypical attention. The utilization of head-mounted eye-tracking techniques provides millisecond-level information about how visual engagement fluctuates around parental input in play contexts, which has great potential to bridge this gap.

One recent line of work has concerned the gaze behaviors of children with autism during live social interaction—these studies use small head-mounted eye-tracking devices to record moment-to-moment gaze behaviors along with the view of the child.

The use of head-mounted eye-tracking has been used in animal studies [20, 22, 33, 46] and has become a popular method among typically-developing adults, children, and infants to study attention and communication behaviors [12, 13, 42, 49]. Head-mounted eye-tracking allows researchers to tap into internal processes that occur in three-dimensional experiences through the participant’s actions and body movements, rather than needing explicit participant responses (e.g., pointing a finger or saying a word) or requiring researchers to provide instructions for a presented computer-based task. However, to date, few studies have utilized head-mounted cameras and/or eye-tracking with children who have autism [34, 48, 50, 51], see also [18, 25] for the review). Furthermore, children with significant social communication difficulties, sensory concerns, low or minimal speaking abilities, and/or challenging behaviors are often excluded from such studies [6, 45]. The limited availability of head-mounted and/or eye-tracking data collected from this population represents a gap in knowledge that, if addressed, may offer insight into potential differences between children whose development follows typical trajectories and those with autism. Expanding the literature basis in this way may increase the generalizability of eye-tracking research results and better reflect the diversity of characteristics among children with autism. Head-mounted eye-tracking methods can also provide insight into critical information about everyday experiences that could inform advances in identifying autism [10, 14, 21] and/or in the development of interventions that target autism-related difficulties [4, 17] both at home and in the community (e.g., daycare and school). Therefore, the pursuit of such data collection is worthwhile despite the method itself presenting practical challenges. To accomplish this, however, researchers need to keep a few important points when conducting head-mounted eye-tracking studies with this population.

The purpose of this manuscript is to share some important pointers to friendly and successful practices that we believe optimize head-mounted camera (specifically head-mounted eye-tracking) data collection. Previous studies have reported varying successful data collection rates for typically developing children, such as 80.00%, 90.90%, and 90.48%, and 47.17% and 56.67% for children with autism [1, 50, 51]. Our lab has reached a successful data collection rate of 93.47% for typically-developing children and 92.68% for children with autism, compatible with or higher than the reported retention rates. The suggested practices – guidelines – outlined in this paper are based on our past and ongoing research with toddlers and children with autism and infants with an increased likelihood of later autism diagnosis. We believe that this may also have potential applicability for collecting these data among young children with other developmental disorders and could be adapted for research with other wearable devices such as electroencephalogram (EEG) and heart-rate monitoring. We will discuss the potential applications to other relevant areas as well as other adaptations researchers may consider utilizing later in this paper.

Please note that this paper uses a combination of person-first and identity-first language, an intentional decision aligning with recent comments put forth by autism researchers, which recognizes the complexities of known and unknown preferences of those in the larger autism community [3, 44].

## Methods

### Participants

For this paper, we selected 41 children with autism and 17 children who are at increased likelihood of later autism diagnosis (e.g., younger siblings of older children with autism) who participated in our study with a head-mounted eye-tracking study in a parent–child object play context. Specifically, children must be under 9 years old and have attempted participation with a head-mounted eye-tracking task in a parent–child play context. Children with autism must also have a diagnosis of autism as defined by the Autism Diagnostic Observation Schedule (ADOS) [26] or the Autism Diagnostic Observation Schedule, Second Edition (ADOS-2) [27]. However, we discuss some relevant anecdotes outside of the population in the later discussion when they are relevant and important. Table 1 summarizes the demographic characteristics of the children with autism sample divided into younger (under 5 years of age; *N* = 22) and older (over 5 years of age; *N* = 19) participants who were between the ages of 1.3 and 8.9 years in addition to the children who are at increased likelihood of later autism diagnosis, who were between the ages of 0.5 and 4.5 years. Families were recruited through local community institutions and social media platforms and were compensated for their participation, including a gift card, local museum pass, and small toy. All families provided informed consent and the study and its procedures were approved by the Institutional Review Board of the university where the research took place.

**Table 1:**
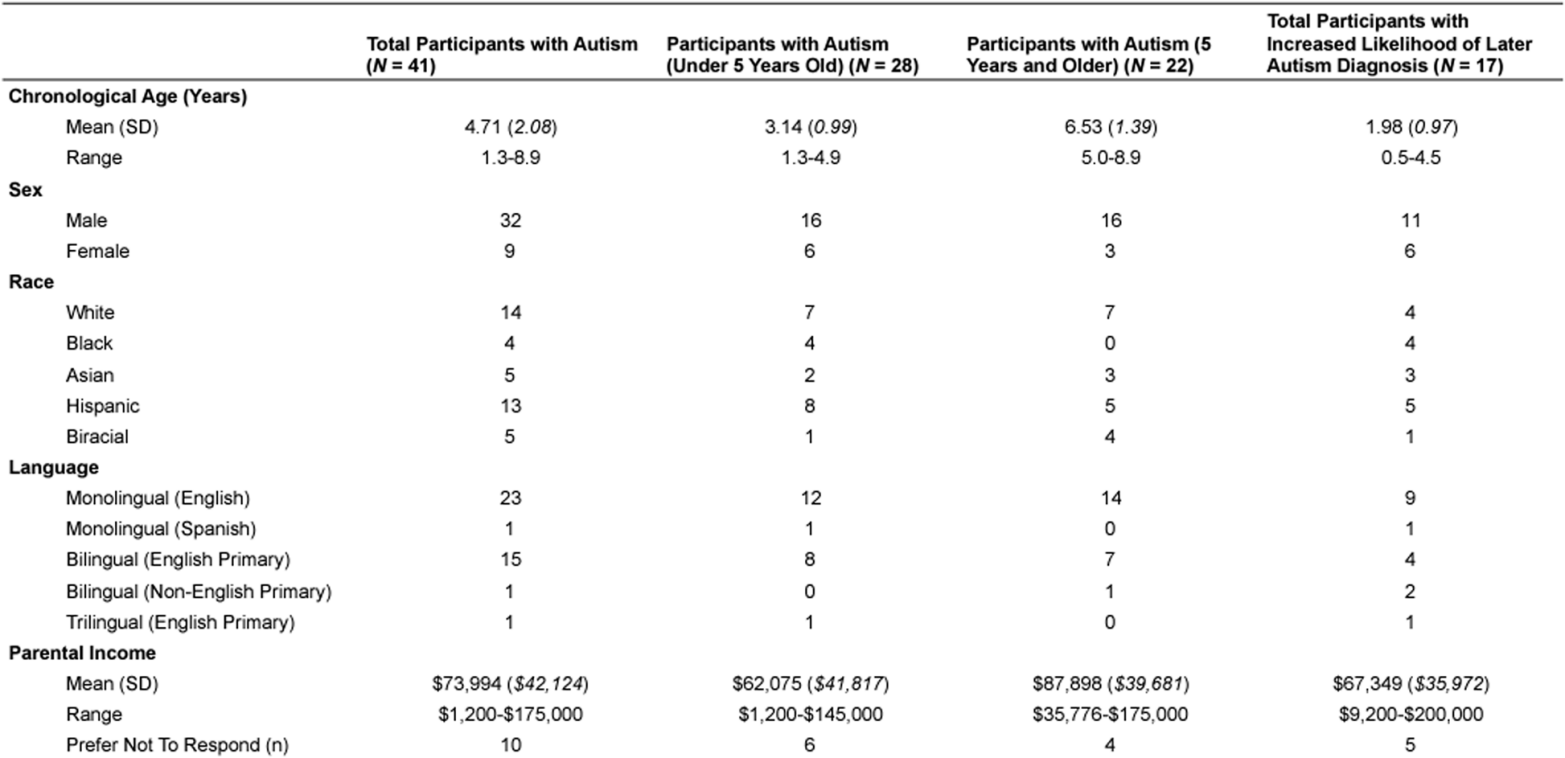
Participant demographic information

### Tasks

ADOS/ADOS-2: All participants with autism in this study exceeded score thresholds consistent with an autism diagnosis on the ADOS or the ADOS-2, which were administered by research-reliable administrators. No auditory or visual impairments were reported by the parents for any of the child participants. The ADOS-2 takes 45 min to 1 h and is appropriate for children 12 months and older who are ambulatory.

Cognitive test: As developmentally- and age-appropriate, participants also participated in one of two cognitive tests, either the Differential Ability Scales—II [9] or the Mullen Scales of Early Learning [29]. On average, the cognitive test takes 20 to 45 min. The DAS-II is appropriate for children 2.5 years through 17 years and 11 months. The Mullen is appropriate for children 0 through 68 months.

Vocabulary test: As developmentally- and age-appropriate, participants also completed the Peabody Picture Vocabulary Test Fourth Edition (PPVT-4) [7] and the Expressive Vocabulary Test Second Edition (EVT-2) [47]. On average, each vocabulary test takes 15 to 30 min for a total of 30 min to 1 h, and both tests are appropriate for children 2.5 years and older.

Head-mounted eye-tracking task: This 5-min task consists of an interactive parent–child play session with a standardized set of eight toy objects. Parents were instructed to play with their children as naturally as they would at home. On average, the head-mounted eye-tracking task takes 20 min to 1.5 h (including set-up, calibration, play, re-calibration, and breaks as needed). Before the play task began, we implemented a series of protocols for placing the head-mounted eye-tracking device on the child and parent participants and calibrating the head-mounted eye-tracking devices for the accuracy of the gaze measurements. This is one of the key elements of the present review and each step is elaborated on in order.

The ADOS/ADOS-2 modules administered, along with the means and standard deviations of the cognitive and language assessment scores, are reported in Table 2.

**Table 2:**
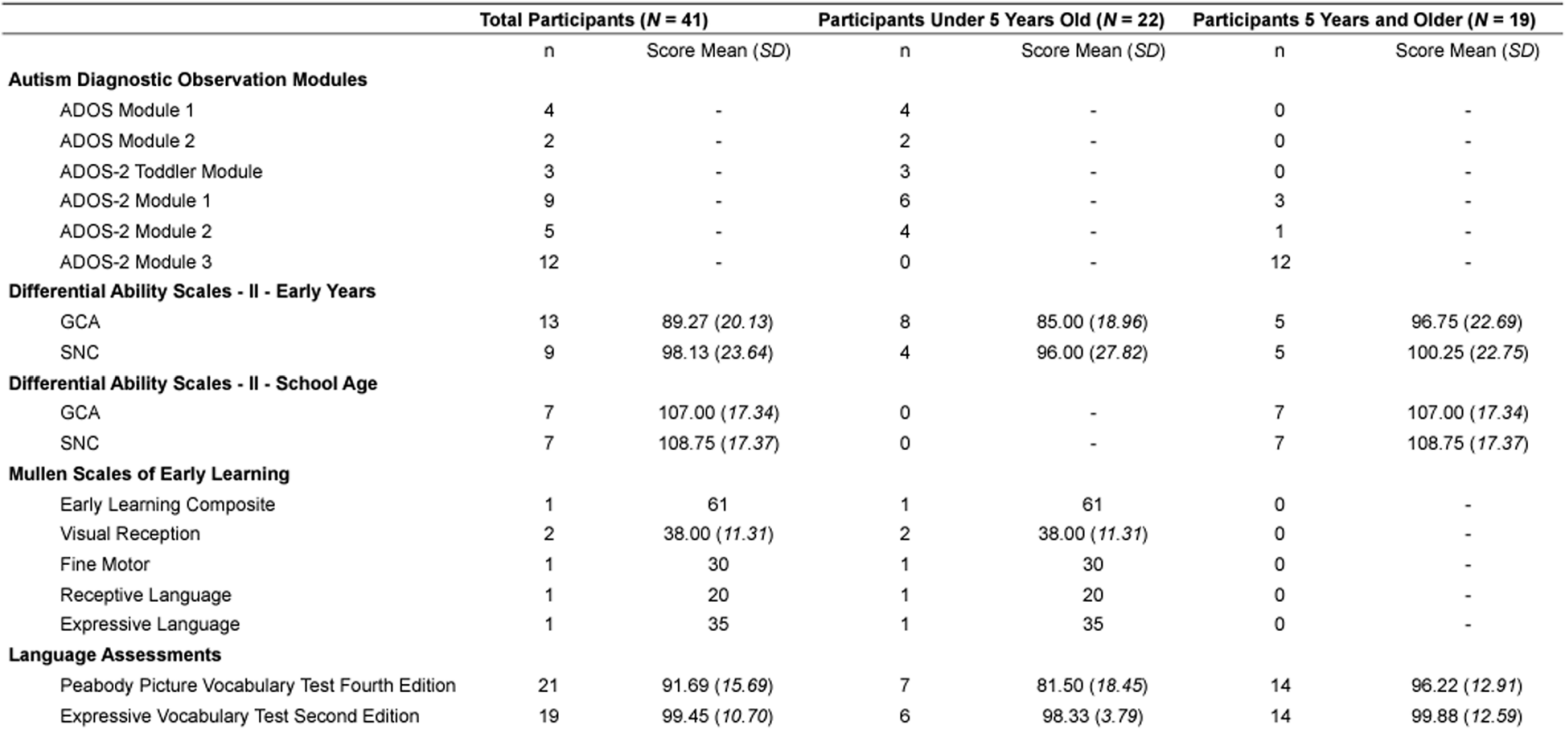
Participant assessment information

### Procedure

#### Screening

Before scheduling participants, parents completed a detailed screening form that included questions about their child’s allergies and dietary restrictions, preferred items and activities that could be used as reinforcers during the study visit, language abilities, and possible sensory considerations. Parents also filled out a developmental history form which included information about their child’s medical history, family medical history, therapy history, and challenging behaviors.

This information allowed our research team to prepare for the individual needs of each child and family that participated, such as gathering preferred items and activities. Detailed information about the study was also provided to families before they agreed to participate, such as a detailed description of all the study tasks, the average time to complete each task, and which research team member would be administering each task. Families were able to discontinue study tasks at any point and were encouraged to ask questions and provide feedback on the process for future studies, which was integrated throughout the continued implementation of study procedures with other families.

#### Pre-visit preparation

**Fig. 1:**
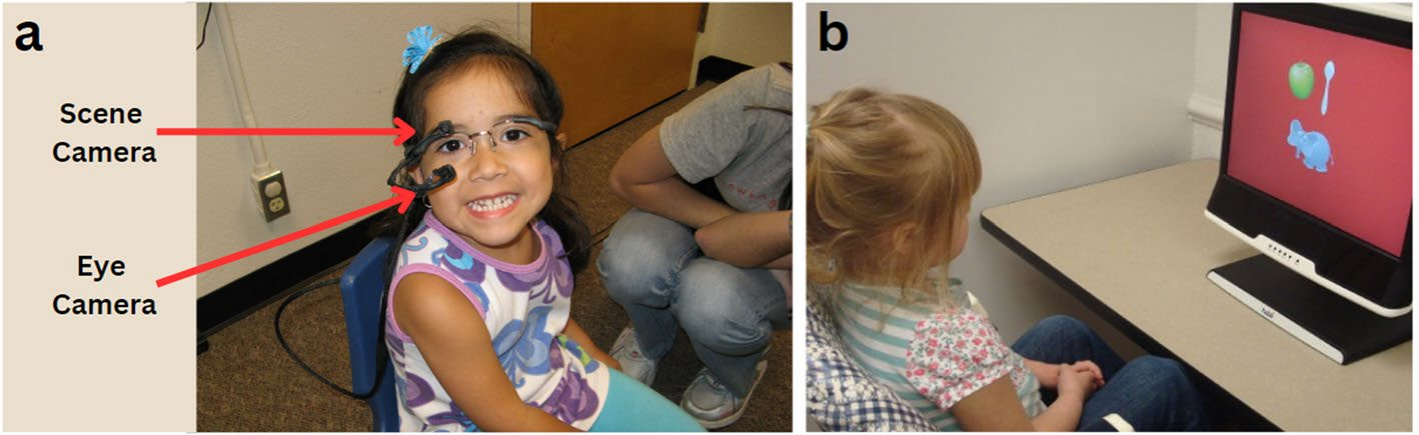
**A** The participant wears the head-mounted eye-tracking device. Corneal reflection is recorded through the eye camera and the gaze location is then measured and superimposed onto the forward-facing view recorded through the scene camera. **B** The participant sits in front of a computer screen. Gaze location is measured through a computer-mounted camera and superimposed onto a recording of the computer display

**Fig. 2:**
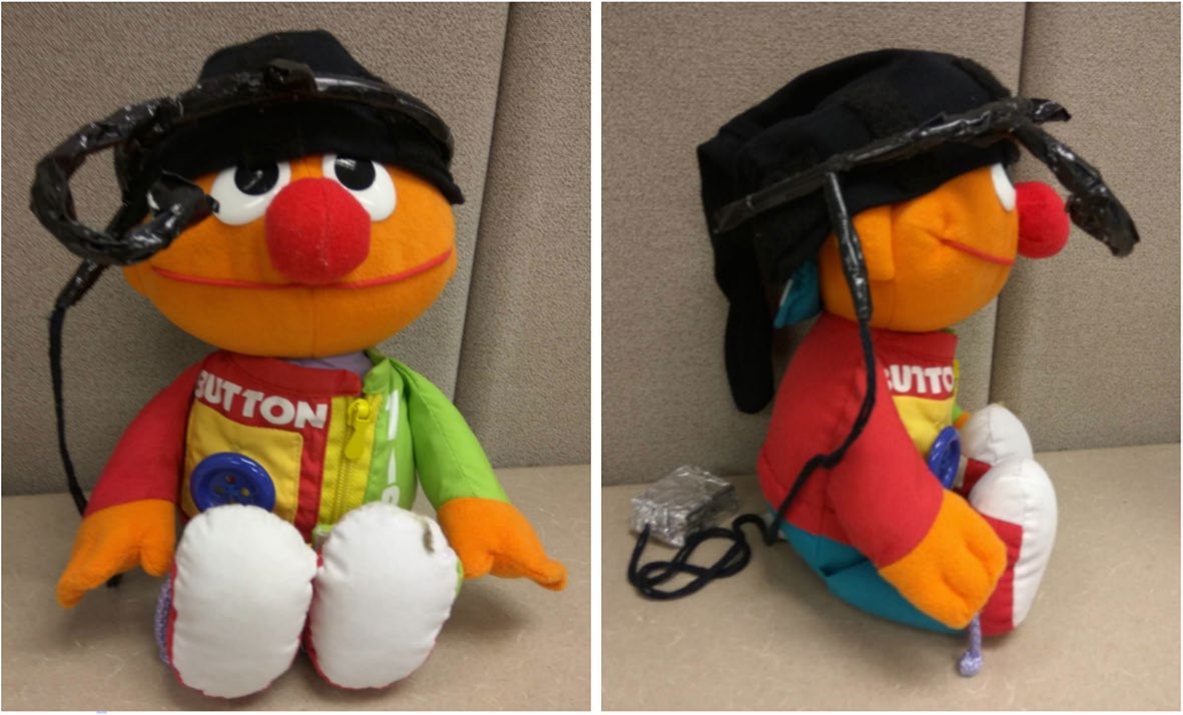
An example image provided to families, showcasing a stuffed animal wearing a mock head mounted eye-tracking device. The following caption accompanied the image for families practicing with the mock head-mounted eye-tracking device at home: “Please place the hat so that the setup resembles the one pictured above. The strip of Velcro should run along the front of the head. The knob on the headband should be placed at the center of the forehead (above the nose) and the eye-tracker (the bent pipe cleaner) should be pointed towards the eye as shown. The box should go over the child’s right shoulder – this will be attached to the back of the chair your child will be sitting in during the play session”

Depending on developmental appropriateness (e.g., the child’s age and language level), parents were encouraged to discuss with their children the head-mounted eye-tracking device they would be asked to wear and the tasks they would be asked to complete when they visited the lab. Pictures and videos of previous participants (with parental permission; see Fig. 1) and stuffed animals wearing head-mounted eye-tracking devices (see Fig. 2) were also provided for families to view at home. Notably, due to the age, cognitive, and linguistic abilities of some of our participants—particularly the younger ones—this was not always possible or beneficial.

Parents were also provided instructions on how to assist their child in wearing a hat for extended periods, to which the head-mounted eye-tracking device would be attached [40]. Examples of this include having the child wear the hat during comfortable routine activities, such as play and meal times, and providing reinforcers to the child for wearing the hat for increasing periods (e.g., 10 s to 30 s to 1 min to 3 min). Our research team also created and offered a mock head-mounted eye-tracking device, and six families opted to practice with this at home before their lab visit. These mock head-mounted eye-tracking devices were made out of lightweight materials, including pipe cleaners, tape, and velcro, which simulated the weight of the actual head-mounted eye-tracking device (see Fig. 1).

An example image provided to families, showcasing a stuffed animal wearing a mock head-mounted eye-tracking device. The following caption accompanied the image for families practicing with the mock head-mounted eye-tracking device at home: “Please place the hat so that the setup resembles the one pictured above. The strip of Velcro should run along the front of the head. The knob on the headband should be placed at the center of the forehead (above the nose) and the eye-tracker (the bent pipe cleaner) should be pointed towards the eye as shown. The box should go over the child’s right shoulder – this will be attached to the back of the chair your child will be sitting in during the play session.”

Parents were asked to bring preferred reinforcers, such as toys and activities, as well as snacks and drinks to the study visit. Parents had the option of splitting the study visit into multiple visits to increase the likelihood of their child completing the study tasks (parent–child play while the child and parent wear the head-mounted eye-tracking devices and the ADOS-2 in addition to the developmentally-appropriate cognitive [DAS-II; Mullen] and language [PPVT-4; EVT-2] tests) successfully and were given flexible scheduling options, including scheduling on evenings and weekends or rescheduling (for example, if the child was unable to sleep the night before the scheduled appointment). Furthermore, study consent and questionnaire forms were sent to parents in advance of their appointment to complete at home if they chose to do so, which also served to reduce the amount of time that children spent downtime in the lab.

#### Testing environment

The study procedure room, which was set up before the child’s visit, contained only essential materials, such as equipment and stimuli, to minimize potential distractions. Reinforcers, such as snacks and toys, were placed out of reach and sight of the child, though were easily accessible by both the research team and the family. This limited the number of objects available for the participant, which limited distraction and the possibility of intentional or incidental self-harm. Minimal examiners (1–3) were present in the lab for each task; one examiner typically set up the equipment and collected the data while the second examiner exchanged testing materials, redirected the child’s attention, and monitored equipment after set up.

To prevent children from becoming distracted by their reflection or activity in the adjacent room, the one-way mirror present in the study procedure room was covered by a curtain. Furthermore, once the family entered the study procedure room, the door was also covered by an additional curtain to limit the possibility of elopement. During the head-mounted eye-tracking task, portions of the room not in use for the task were blocked by a third curtain, preventing the child from seeing the computer monitoring equipment or materials for later tasks. Furthermore, for tasks that did not require their participation, parents made the decision whether to be present in the room, based on their perspective of how well the child would perform with their parent being present or absent.

Standardized office lighting levels (approximately 300 lx) were used during the study tasks to decrease the likelihood of children becoming overstimulated. The standard lighting had the additional benefit of making it easier for the eye-trackers to capture the pupil and corneal reflection, which decreased the amount of time that the child participants had to wear and tolerate the eye-tracker device.

#### Visit—waiting room

Upon entering the lab, children were permitted to play freely with a variety of different toys in a non-testing, child-friendly waiting room while the family completed consent forms nearby. This served three purposes: 1) to build rapport with a researcher who played alongside the child, making sure not to place demands on the child during this time, and not forcing the child to engage with them if the child did not wish to do so, 2) acclimating the child to the novel laboratory environment, and 3) acclimating the child to the hat (which the head-mounted eye-tracking device would be attached to) that would be used during the head-mounted eye-tracking task. This period lasted anywhere from 5 to 20 min.

To aid in acclimation, families (and their children when developmentally appropriate) were given the option to have the hat and/or head-mounted eye-tracking device placed on the child by a researcher, by a parent, or by the child themselves. Children were also able to pick a hat from a variety of different colors and sizes and were allowed to touch the hat before placement [40]. Our research team was encouraged to provide choices whenever possible to the child, such as “Do you want to wear the red hat or the blue hat?”, but were instructed to avoid yes / no questions such as “Do you want to wear the hat?”. For children who were reluctant to wear the hat, researchers and parents demonstrated wearing the hats themselves before placing the hat on the child’s head; for children who continued to demonstrate reluctance, the parents and/or researchers also wore hats alongside the child to encourage them to leave the hat on their head. Older children were also given the option to wear a pair of glasses to which the head-mounted eye-tracking device was attached instead of having the eye-tracker attached to a hat.

In some cases, families indicated before their visit that their children may become distracted if they were exposed to the waiting room before the study tasks. For these visits, researchers hand-picked a few toys that matched the child’s preferred interests to place in the study procedure room. The family entered the lab through the back entrance, bypassing the waiting room (which stayed out of sight of the child), and went directly to the study procedure room. The child and researcher then played at the table that would be used for study tasks while the parent filled out the consent forms.

#### Visit—study procedure room

If necessary, children were allowed to bring a toy from the waiting room to accompany them to the study procedure room to assist in transitioning to the study procedure room. Upon entering the pre-prepared study procedure room, the child and the parent sat at a child-sized table, during which the child was given the choice of which side of the table to sit at, as well as the choice of whether to sit in the red or the blue child-sized chair.

Typically, 1 to 2 researchers were present to minimize the child’s exposure to strangers and decrease the possibility of the child becoming overwhelmed; in cases where the family visited the lab more than once (e.g., longitudinal project), efforts were made to have the same researchers present at each visit.

Between study tasks, families and their children were asked if they would like to take a break. Breaks included going to the bathroom, eating a snack or lunch, getting a drink, taking a walk, or engaging in preferred activities without demands being placed. These individualized breaks generally lasted between 5 and 20 min and could take place within the study procedure room, in the playroom, or outside of the lab. Families were also informed that they may ask for a break at any point in the middle of study tasks. Though these breaks extended the length of the study visit, they reduced the likelihood of the child engaging in challenging behaviors and therefore may have ultimately reduced the time testing would have taken should the child have been continuously tested.

#### Visit—head-mounted eye-tracking task

Once the parent and child were settled in the study procedure room, a researcher first placed and calibrated – the procedure for taking the geometric characteristics of a participant’s eyes into account to optimize the accurate gaze point calculations – the parent eye-tracker while a second researcher played with the child [11, 40]. This reduced the amount of time that children had to tolerate wearing the head-mounted eye-tracking device. The head-mounted eye trackers used in this study were purchased from Positive Sciences, Inc. and consisted of two small cameras and an infrared light-emitting diode (LED) which weighed 51 g in total. One camera faces the participant’s right eye, recording the pupil movements and the corneal reflection, while the head-mounted camera placed on the forehead records the visual field from the participant’s perspective (FPV: 54.4° horizontal by 42.2° vertical).

##### Putting head-mounted eye-tracker on child

Once the parent eye-tracker set-up was complete, one researcher focused on engaging the child in play or other preferred activities while the second researcher placed the eye-tracker on the child’s head, providing something for the child to focus on other than the placement of the eye-tracker. Parents were also encouraged to assist in distracting their child during the child’s eye-tracker placement, such as playing with toys, holding hands, or singing a song. When children were reluctant to wear the head-mounted eye-tracking device, a variety of strategies were employed to help acclimation. Researchers and/or parents placed the hat and/or head-mounted eye-tracking device on themselves to demonstrate to the child what they would be wearing. Some families also utilized a mirror or phone camera to show their children the items being placed. When developmentally appropriate, the computer screen with live videos of the child’s eye and point of view was shown to the child, and researchers explained the purpose of the device. Children were also verbally assured that their eyes would not be touched and were encouraged to let researchers and their parents know if the hat or head-mounted eye-tracking device became uncomfortable or if they would like to switch hats or devices when the child was verbally and/or developmentally able to do so. If a child became overly distracted by the head-mounted eye-tracking device and tried to touch, play with, or move the camera, researchers and parents gently physically redirected their hands to preferred items and reminded the child not to touch the camera. Extra tape and velcro were applied to the head-mounted eye-tracking device and hat if necessary, to further secure equipment and prevent removal during calibration and the play session. Positive feedback, such as verbal praise, access to preferred items and/or activities, and high-fives were regularly provided to the child during this process, particularly after the eye-tracker was placed.

##### Calibration procedure with child

Once the head-mounted eye-tracker was placed on the child’s head, research assistants conducted a calibration procedure to ensure the appropriate accuracy of the gaze location tracking relative to the scene through the dual recording of the child’s corneal reflection and their first-person view. During the child’s calibration session, a research assistant directed the child’s attention to a series of nine targets on a flat board. A variety of different methods were used to direct children’s attention to the different calibration points, beginning with what was most developmentally appropriate and continuing until the child was most responsive: verbal instructions, pointing with a finger, pointing with an item (e.g., a pen), holding a preferred item in front of the calibration point (e.g., a small toy carrot), and shaking a preferred noise-making item in front of the calibration point (e.g., a rattle toy). In some cases, such as when children were overly shy, parents assisted in directing the child’s attention to each point rather than the researcher doing so. A second researcher held the calibration board in place and ensured the child was looking at the target by monitoring the computers and the child’s gaze direction. Again, verbal praise was used, particularly after the child successfully attended to a calibration point. This procedure generally took less than 5 min.

After this first calibration session, parents were instructed to play with their child as naturally as possible for the standardized duration of the study task (320 s) using a set of eight standard toys that were used for all participants enrolled in the study. During the head-mounted eye-tracking task, it was helpful for some children to use a sensory or comfort item (such as a blanket), have a researcher begin a timer to visually track how long the child would wear the head-mounted eye-tracking device, and/or listen to music while the head-mounted eye-tracking device was placed and calibrated.

Once this head-mounted eye-tracking play session was complete, a second head-mounted eye-tracking calibration session was conducted before the eye-trackers were removed following the same procedures as described prior with one exception. The child's eye-tracker was calibrated and removed before the parent’s eye-tracker was calibrated and removed; this further reduced the amount of time that the child had to tolerate the device. Again, positive feedback was used, particularly after the child successfully attended to a calibration point.

Table 3 summarizes the number of visits, the types of reinforcers, and some of the supports used for our participants as described in this section.

**Table 3:**
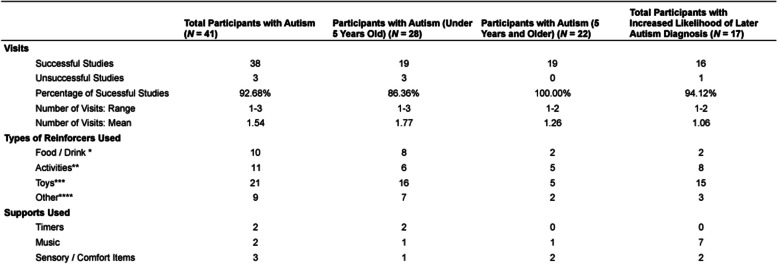
Information about experimental visits for participants

* Food and drink included Cheerios, fruit gummies, Cheez-Its, pretzels, M&Ms, Skittles, McDonald's, lollipops, Fruit Loops, milk, soda, and crackers

** Activities included coloring, screen time (videos or games on a phone or tablet), taking a walk, and reading a book

*** Toys included stuffed animals, rattles, trucks, bubbles, books, puzzles, pop-up toys, and dolls

**** Other reinforcers included hugs, a sensory blanket, promises of a trip to the museum and/or zoo, and turning off and on a light switch

#### Challenges encountered during study visits

Despite best efforts, some study visits were unsuccessful; that is, study tasks could not be completed with 3 of 41 participants with autism, all of whom were in the younger age range (i.e., under five years old). This resulted in an overall success rate of 92.68% for the head-mounted eye-tracking task among all participants with autism: 86.36% among younger participants and 100.00% among older participants. Of the six families who opted into practicing with the mock head-mounted eye-tracker at home, five children completed the eye-tracking task on their first visit. We did not find any differences in the types and frequencies of behaviors among these children compared to children who did not practice with the mock head-mounted eye-tracker at home. The head-mounted eye-tracking task could not be completed with 1 of 17 participants at an increased likelihood of later autism diagnosis, resulting in an overall success rate of 94.12%. There were no substantial differences in demographic or clinical information between the participants who completed the head-mounted eye-tracking task and the participants who did not. Even for successful visits, however, challenging behaviors were not uncommon, as depicted in Table 4. If the child removed the eye-tracking device, the play session was immediately paused, the eye-tracker location was corrected, and the child was re-calibrated accordingly. On average, each child contributed 8,757 frames (SD = 689) of data per camera for annotation and analyses—approximately 90.37% of the play session. These numbers are consistent with other studies of similar play session lengths [41, 42].

**Table 4:**
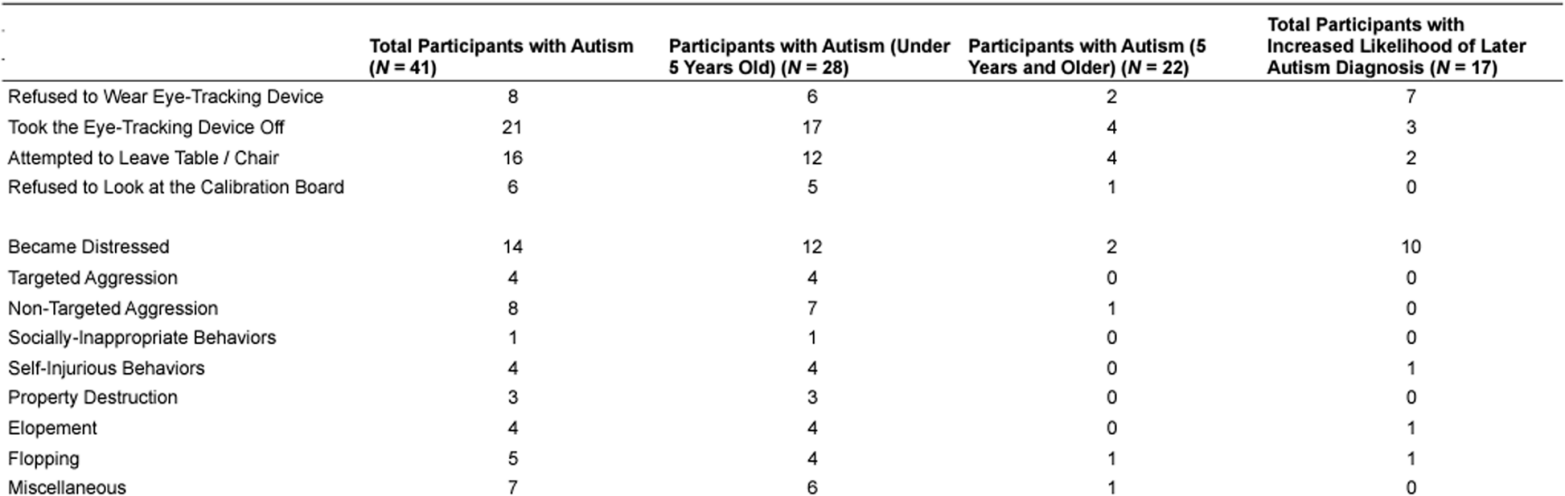
Frequency of participants who engaged in behaviors during the experimental visit

For example, some participants did not wish to wear the head-mounted eye-tracking devices and/or the hats, attempted to leave the chair and/or table, became distressed, refused to look at the calibration board by turning around or staring at the table, or engaged in other challenging behaviors, such as aggression or flopping. When such behaviors occurred, researchers made decisions of study termination through careful observation of the child and by following the parents’ guidance. Young children with autism demonstrate individual differences in their expressions of distress, therefore making it more difficult to determine what the distress level may mean relative to their everyday experiences. This is another point that highlights the importance of the collaborative framework in which we conduct studies alongside our parent participants. In our work, parents and researchers closely work together to monitor the tolerance level of each participant and end study tasks before participants reach their tolerance threshold or engage in particularly challenging behaviors, such as aggression or self-harm. In some cases, families completed the study measures across two or more visits; however, in other cases (e.g., the three described above), participation was discontinued.

## Discussion

In this paper, we have offered a series of approaches our lab has used to gather data from young children with autism and increased likelihood of later autism diagnosis of various ages, symptom severity, and language and communication backgrounds by using the head-mounted eye-tracking method. We believe that the general approaches described here can also be extended to other types of data collection, such as EEG and heart-rate monitoring, as well as to data collection from children with other disorders and disabilities. For example, in addition to working with the 41 autistic children and 17 children with increased likelihood of later autism diagnosis described in this paper, our lab has successfully collected head-mounted eye-tracking data from 12 deaf children, 3 children with language disorders, and 1 child with an attention disorder between the ages of 7 months and 4.5 years using the methods described here. Our lab has also successfully collected head-mounted eye-tracking data from typically developing children, ranging from infants to elementary age, and focused on the strategies that are specifically effective for children with autism and other disabilities and developmental backgrounds in the present paper.

In our studies, we use a variety of techniques to support the data collection described in this manuscript. Desensitization training, such as “practice” with nonfunctional equipment and stepwise procedures to acclimation has been successfully used across different methods of data collection among individuals with autism but requires a high amount of personalization to individual labs, study tasks, and equipment [36, 37, 43]. Through our studies, we have been trying out reported strategies to attach electrodes for conventional clinical EEG recordings from infants and toddlers [5, 6, 19] and developing new ones that are effective for our sample of children with autism and increased likelihood of later autism diagnosis to promote successful head-mounted eye-tracking data collection. One such desensitizing procedure includes offering families the opportunity to practice at home with mock head-mounted eye-tracking devices, utilizing stepwise acclimation for wearing the head-mounted eye-tracking device (comfortability with the environment first followed by the hat then the head-mounted eye-tracking device) and seeing demonstrations by researchers and families of wearing the head-mounted eye-tracking device. Strategies grounded in behavioral science have been useful in others’ work, such as in the handling of challenging behaviors, the use of antecedent control, and the application of positive consequences [43]. In our studies, we also use behavioral science techniques such as modeling wearing the head-mounted eye-tracking device, reinforcing appropriate responses (e.g., keeping the head-mounted eye-tracking device on), and breaking down study tasks into incremental steps. Importantly, we have worked to partner with parents to maximize the flexibility and individualization of our study procedures while adhering to the fidelity of our protocols for gathering data. The importance of tailoring interventions to fit specific children and families has been recognized in providing care for young children with developmental concerns [35], and we believe that the concept of “flexibility within fidelity” originally proposed for both intervention and dissemination efforts [23] also applies to data collection procedures. Preparing young children for participation in head-mounted eye-tracking studies has, in our experience, been fruitfully aided by thoughtfully considering their preferences, strengths, and needs in domains ranging from the day and time of the appointment to the toys selected for their play. One important issue relevant to the individualized task context in the research domain is to create confounds. For example, the individualized timings of the breaks we often employ may uniquely impact age-related findings, as older participants in general required less break time than our younger participants. This point needs to be carefully considered in the relation to the research questions and hypotheses.

While our current work has been successful in ensuring that young children with autism and increased likelihood of later autism diagnosis are comfortable with the head-mounted eye-tracker and that the majority of children complete the task, the data presented here highlight that not all of our participants were able to complete the head-mounted eye-tracking task despite multiple attempts and application of the strategies. In general and not surprisingly, we found that our older participants completed the study in fewer visits, engaged in less challenging behaviors, tolerated the head-mounted eye-tracking gear more easily, and required fewer reinforcers, breaks, and supports to be used to maintain attention and tolerance. This may be because older participants had more experiences in other structured environments where similar demands are placed (such as school) or higher communication and language skills than the younger participant group. Additionally, older participants may have had more experiences wearing hats and glasses, which increased the likelihood of being able to tolerate wearing the head-mounted eye-tracking device or may have more easily established rapport with our research team. For infants, toddlers, and young children, it may be more optimal to encourage multiple study visits and increase the time in which children acclimate to the lab environment—however, such practices must be mediated by considerations of substantive time and financial commitments by both families and their children in traveling to and from research settings.

There are a variety of other possible and viable adaptations that previous researchers have used in other neuroscience studies focused on children with autism that researchers should consider utilizing to assist head-mounted eye-tracking data collection. For example, EEG and polysomnography (PSG) protocols have used mirrors to allow children to see the EEG cap being applied and token boards to visualize reinforcer schedules to increase the likelihood of successful scans among children with neurodevelopmental disabilities, including autism [32]. Magnetic resonance imaging (MRI) studies have used behavioral science strategies and mock MRI scan training among autistic children aged 9 to 13 years [30], while magnetoencephalography (MEG) studies have integrated visual stories and walking interviews with autistic children aged 8 to 12 years, with a success rate of 81% for completed tasks and 74% for usable data [24]. MRI connectivity studies involving children with autism ages 7–17 years who have low verbal and cognitive scores have taught families anxiety reduction techniques, had children view pre-determined “relaxing” images and videos, and had children wear noise-canceling headphones [16]. In other cases, research teams had board-certified behavior analysts who created personalized plans for each participant to optimize data collection processes [30]. Indeed, in our studies, we have found that collaboration between basic and applied scientist members of our team is helpful in continually refining procedures to consider individual characteristics. Finally, researchers should consider consulting other published data collection guidelines for children with autism, such as EEG and computer eye-tracking protocols [38, 45]. These methods provide foundations for increasing data collection from young autistic children and incorporate children across the spectrum who differ in language, cognitive, and other abilities to reflect the heterogeneous nature of symptom presentation and severity within this neurodevelopmental diagnosis. Such practices will increase the generalizability of results for this diverse population and guide the development of future research studies investigating early gaze behaviors.

## Conclusion

Though collecting quality data using head-mounted eye-tracking devices from young children with autism and increased likelihood of later autism diagnosis has its challenges, there are many benefits, and our work along with other recent studies suggest that this can be successfully undertaken. Data that emerge from such studies may have implications for increasing our knowledge of the everyday experiences of autistic children and offering insight into attentional development among this population. Ultimately, this line of work holds promise for improving screening and diagnostic services. As head-mounted eye-tracking methods continue to improve amongst this growing population, we expect that further micro-behavioral analysis of children’s perceptual and communication experiences during day-to-day life will be possible, opening up new avenues not only for basic science but also for potential applications for clinical and applied work.

## Data Availability

The datasets used during the current study are available from the corresponding author upon reasonable request.
